# Drug versus vaccine investment: a modelled comparison of economic incentives

**DOI:** 10.1186/1478-7547-11-23

**Published:** 2013-09-08

**Authors:** Stéphane A Régnier, Jasper Huels

**Affiliations:** 1 Université de Neuchâtel, Pierre-à-Mazel 7, Neuchâtel CH-2000, Switzerland; 2 Novartis Vaccines & Diagnostics AG, Lichstrasse 35, Basel, 4056, Switzerland

**Keywords:** Incentives, Vaccines, Drugs, Research and development, Investment, Net present value

## Abstract

**Background:**

Investment by manufacturers in research and development of vaccines is relatively low compared with that of pharmaceuticals. If current evaluation technologies favour drugs over vaccines, then the vaccines market becomes relatively less attractive to manufacturers.

**Methods:**

We developed a mathematical model simulating the decision-making process of regulators and payers, in order to understand manufacturers’ economic incentives to invest in vaccines rather than curative treatments. We analysed the objectives and strategies of manufacturers and payers when considering investment in technologies to combat a disease that affects children, and the interactions between them.

**Results:**

The model confirmed that, for rare diseases, the economically justifiable prices of vaccines could be substantially lower than drug prices, and that, for diseases spread across multiple cohorts, the revenues derived from vaccinating one cohort per year (routine vaccination) could be substantially lower than those generated by treating sick individuals.

**Conclusions:**

Manufacturers may see higher incentives to invest in curative treatments rather than in routine vaccines. To encourage investment in vaccines, health authorities could potentially revise their incentive schemes by: (1) committing to vaccinate all susceptible cohorts in the first year (catch-up campaign); (2) choosing a long-term horizon for health technology evaluation; (3) committing higher budgets for vaccines than for treatments; and (4) taking into account all intangible values derived from vaccines.

## Background

It has been argued that the vaccines market is not attractive to manufacturers [1]. Even with the successful launches of vaccines against pneumococcal and human papilloma virus diseases and pandemic influenza, vaccines’ share of the global medicines market remains marginal at approximately 3% (2010 figures) [2,3]. Historically, manufacturers have preferred to invest in potential blockbusters and the number of manufacturers producing vaccines in the USA dropped from 37 to 10 between 1967 and 2002 [1,4,5]. Currently, four-fifths of the market is held by only five manufacturers [3]. As a consequence, investment in vaccines is relatively low, with manufacturers only spending $750 million on research and development (R&D) for vaccines in 2000 compared with $26.4 billion for pharmaceuticals [1,6]. One of the factors explaining the situation is low pricing, driven by the fact that not all of the intangible value may be taken into account when vaccines are evaluated [5-8].

We therefore developed a mathematical model to elucidate whether the methodologies currently used to evaluate new technologies favour drugs over vaccines. We modelled the decision-making process of manufacturers when deciding to invest in a vaccine or a drug to combat a disease that affects children, by analysing the interactions between manufacturers and payers. In this article, we report the model and also discuss the reasoning behind and potential implications of each finding. Since the major vaccines manufacturers (GlaxoSmithKline, Merck, Novartis, Pfizer, Sanofi) also produce treatments, the question is particularly relevant and important. It should be highlighted that (i) the model intends to assess the *relative* attractiveness of vaccines compared to drugs and does not intend to evaluate the *absolute* attractiveness of the vaccines market, and that (ii) the article only explores the economic arguments (scientific arguments are only evoked where relevant). Please note that economic arguments are not the only elements under consideration when a decision is made. For instance, a risk-free inactivated polio vaccine would not be recommended if cost-effectiveness was the only decision criterion [7].

## Methods

### The model

The model describes investment in technologies to combat a disease that affects children from the perspectives of the manufacturer and the regulator or payer. The disease impacts *n*_*c*_ cohorts of children (i.e., from age 0 to *n*_*c*_*–* 1) (Table 1 provides a summary of terms used in the model). The larger *n*_*c*_ is, the more widespread the disease. Each cohort size is normalized to 1 (or 100%). The probability of becoming sick is uniformly distributed across cohorts and is equal to *s* per year in each cohort.

**Table 1:** Summary of terms used in the model

**Term**	**Definition**
***B***	Budget threshold (regulator)
***CA***_***ji***_	Cost avoided with technology *j* in year *i*
***c***_***c***_	Annual cost of the disease per cohort before introduction of a new technology
***C***_***ji***_	Cost associated with developing and selling technology *j* in year *i*
***d***	Number of years after launch used for economic evaluations by the manufacturer
***E***_***ji***_	Incremental quality-adjusted life-years gained from technology *j* in year *i*
***j***	Technology indicator(*j* = 0 for treatment; *j* = 1 for prevention)
***l***	Number of years required to develop technology before launch
***M***_***ji***_	Gross margin for technology *j* in year *i*
***t***	Time horizon in years used for economic evaluation (regulator)
***n***_***c***_	Number of cohorts susceptible to the disease
***P***_***ji***_	Price of technology *j* in year *i*
***Q***_***ji***_	Quantity (demand) for technology *j* in year *i*
***r***_***m***_	Discount rate used by manufacturer to evaluate investment
***r***_***r***_	Discount rate used by regulator to evaluate new technologies
***s***	Annual probability of children in susceptible cohorts becoming sick
***S***_***ji***_	Sales of technology *j* in year *i*
***ψ***_***t***_**(*r*)**	Discounting factor in the *t*^th^ year, if the discount rate is *r*

The model consists of two players: the manufacturer who decides to invest in the technology and the regulator/payer who sets the price and the demand by determining the individuals eligible for treatment. The timing of the game is as follows: first, the regulator announces how new health technologies are to be evaluated and used; the manufacturer then decides to invest in a treatment (drug) or in prevention (vaccine). The regulator can decide to use the potential vaccine to either (i) vaccinate infants in their first year of life (routine vaccination); or (ii) vaccinate infants in their first year of life and vaccinate all susceptible cohorts (routine vaccination plus catch-up). The efficacy of the drug and the vaccine are assumed to be the same. If all sick individuals are treated (or all susceptible cohorts are vaccinated), the current disease costs are assumed to be eliminated. There is no asymmetry of information between the regulator and the manufacturer.

We will now describe each player, and their objective and strategy.

#### The regulator

The regulator can set the price of new technologies based on two potential criteria: (i) budget impact; or (ii) cost-effectiveness. It should be noted that, among other criteria, the French authorities usually apply budget impact-related arguments when negotiating the price of new technology with manufacturers, while the National Institute for Health and Clinical Excellence for drugs in England and Wales and Joint Committee on Vaccination and Immunisation for vaccines in the United Kingdom use a cost-effectiveness framework [9].

In the budget-impact framework, the regulator is willing to accept a new technology provided that incremental costs are below a certain annual budget threshold *B*_*j*_, where *j* = 0 for treatment and *j* = 1 for prevention. In the model, the allocated budget could be different for treatment and prevention. Therefore, the new technology is accepted if:

(1) $$\sum_{i=1}^{t}\frac{Q_{\mathrm{ji}}P_{\mathrm{ji}}-CA_{\mathrm{ji}}-B_{j}}{{\left(1+r_{r}\right)}^{i}}\leq 0$$

where *Q*_*ji*_, *P*_*ji*_, and *CA*_*ji*_, are the quantity, price paid, and cost avoided in year *i* for product *j*; *r*_*r*_ is the discount rate used by the regulator; and *t* is the time (in years) used in the economic evaluation of the new technologies by the regulator (i.e., the time horizon) (see Table 1).

In the cost-effectiveness framework, the ratio of incremental costs and humanistic benefits is evaluated and compared with a threshold λ. The new technology is accepted if:

(2) $$\frac{\sum_{i=1}^{t}\frac{Q_{\mathrm{ji}}P_{\mathrm{ji}}-CA_{\mathrm{ji}}}{{\left(1+r_{r}\right)}^{i}}}{\sum_{i=1}^{t}\frac{E_{\mathrm{ji}}}{{\left(1+r_{r}\right)}^{i}}}\leq \lambda$$

where *E*_*ji*_ is incremental quality-adjusted life-years gained in year *i* for product *j*.

In the rest of the article, we will only use the budget-impact framework as equation (2) can be considered to be a particular case of equation (1) with:

$$B_{j}=\frac{\lambda \;\sum_{i=1}^{t}\frac{E_{\mathrm{ji}}}{{\left(1+r_{r}\right)}^{i}}}{\sum_{i=1}^{t}\frac{1}{{\left(1+r_{r}\right)}^{i}}}$$

#### The manufacturer

The manufacturer chooses to develop the vaccine rather than the drug if the expected profits of the vaccine exceed those of the drug (3a) and are positive (3b):

(3a) $$E\;\left[\sum_{i=1}^{l+d}\frac{\left(S_{1i}M_{1i}-C_{1i}\right)-\left(S_{0i}M_{0i}-C_{0i}\right)}{{\left(1+r_{m}\right)}^{i}}\right]\geq 0$$

and

(3b) $$E\;\left[\sum_{i=1}^{l+d}\frac{S_{1i}M_{1i}-C_{1i}}{{\left(1+r_{m}\right)}^{i}}\right]\geq 0$$

where *S*_*ji*_ are the sales, *M*_*ji*_ the gross margins, and *C*_*ji*_ the cost associated with developing, marketing and selling the health technology *j* in year *i*; *r*_*m*_ is the discount rate used by the manufacturer; *d* is the number of years after launch used for the economic evaluation; *l* is the number of years required to develop the technology before launch; and *l* + *d* is the total number of years under consideration in the economic evaluation by the manufacturer. The condition (3b) ensures that the net present value of the vaccine’s profits is positive.

### Assumptions and definitions

There are a number of simplifying assumptions in the model: (i) The regulator sets a unique price during the game (i.e., *P*_*ji*_ *= P*_*j*_). (ii) Once a regulator defines the individuals eligible to receive the new health technology, all eligible individuals receive it and are reimbursed by the payer. In other words, the adoption is immediate and the coverage rate is 100%. (iii) The regulator and the manufacturer use the same time horizon to make a decision after the new product’s launch (i.e., *d* = *t*). (iv) The gross margins, costs of development, marketing costs, patent protections, and probabilities of success are assumed to be identical between the technologies (i.e., *C*_*1i*_ = *C*_*0i*_). In particular, this implies that a vaccine is not more or less scientifically difficult to develop than a curative treatment. This assumption allows specific focus on the impact of the evaluation framework and not on the products’ characteristics. The impact of this assumption is discussed later in this article. (v) The annual cost of the disease (*c*_*c*_) is constant for each cohort. (vi) The number of years considered for evaluation exceeds the number of cohorts (i.e., *t* ≥ *n*_*c*_).

*Definition for discounting factor in the t*^*th*^*year:*

(4) $$\psi_{t}\left(r\right)=\frac{1}{{\left(1+r\right)}^{t}}$$

Based on Assumptions iii and iv, the manufacturer chooses the technology with the higher discounted revenues after launch, and condition (3) can thus be rewritten as:

(5) $$\sum_{i=1}^{t}\frac{S_{1i}-S_{0i}}{{\left(1+r_{m}\right)}^{i}}\geq 0$$

## Results and discussion

The maximal justifiable prices accepted by the regulator and, therefore, the maximal revenues generated by the manufacturer, are summarised in Table 2. All proofs can be found in Additional file 1.

**Table 2:** Evaluation of treatment and prevention

	**Treatment (*j*** **= 0)**	**Prevention (*j*** **= 1)**	
Parameter	(A)	Routine and catch-up (B)	Annual routine only (C)
Demand (*Q*_*ji*_)	*sn*_*c*_	*n*_*c*_ if *I* = 1	1
		1 if *I* > 1	
Cost avoided (*CA*_*ji*_)	*c*_*c*_*n*_*c*_	*c*_*c*_*n*_*c*_	i^.^c_*c*_ if *I* ≤ *n*_*c*_
			*c*_*c*_*n*_*c*_ if I > *n*_*c*_
Price (*P*_*j*_)	1/(*sn*_*c*_)·(*c*_*c*_*n*_*c*_ + *B*_*0*_)	$\frac{1+r_{r}-\psi_{t-1}\left(r_{r}\right)}{1+n_{c}r_{r}-\psi_{t-1}\left(r_{r}\right)}\cdot \left(c_{c}n_{c}+B_{1}\right)$	$B_{1}+c_{c}\cdot \frac{1+r_{r}-\psi_{n_{c}-1}\left(r_{r}\right)-n_{c}r_{r}\psi_{t}\left(r_{r}\right)}{r_{r}\left(1-\psi_{t}\left(r_{r}\right)\right)}\;$
Annual sales (*S*_*ji*_)	(*c*_*c*_*n*_*c*_ + *B*_*0*_)	*P*_*1*_·*n*_*c*_ if *i* = 1	$B_{1}+c_{c}\cdot \frac{1+r_{r}-\psi_{n_{c}-1}\left(r_{r}\right)-n_{c}r_{r}\psi_{t}\left(r_{r}\right)}{r_{r}\left(1-\psi_{t}\left(r_{r}\right)\right)}\;$
		*P*_*1*_ if *i* > 1	
Total revenues^a^	$\frac{1-\psi_{t}\left(r_{m}\right)}{r_{m}}\cdot \left(c_{c}n_{c}+B_{0}\right)$	$\frac{1-\psi_{t}\left(r_{r}\right)}{r_{m}}\cdot \frac{1+r_{r}}{1+r_{m}}\cdot \frac{1+n_{c}r_{m}-\psi_{t-1}\left(r_{m}\right)}{1+n_{c}r_{r}-\psi_{t-1}\left(r_{r}\right)}\cdot \left(c_{c}n_{c}+B_{1}\right)$	$\frac{1-\psi_{t}\left(r_{m}\right)}{r_{m}}\cdot P_{1}$

^a^Manufacturers’ revenues discounted from the time of launch until the end of the evaluation. See Table 1 for definitions of symbols.

### Price

Unlike the price of treatment, the price of a vaccine is independent of *s*, the annual percentage of children in susceptible cohorts falling ill. This is because the number of individuals receiving a vaccine is independent of *s*, and only the overall cost of the disease (and not the cost per sick individual) matters when the price is evaluated.

The price of the treatment is independent of the treatment horizon used for the evaluation because the benefits and costs are the same for each year. As 1 + *n*_c_*r*_*r*_ ≥ 1 + *r*_*r*_ and ψ_t-1_(*r*)decreases with *t*, the price of the routine plus catch-up vaccine increases with *t*, i.e., the longer the time horizon chosen by the regulator, the higher the justifiable price. Similarly, the economically justifiable price of the routine programme increases with *t*. The price of the routine plus catch-up vaccine and the price of routine vaccine decrease with the regulator discount rate, *r*_*r*_.

Assuming *B*_*0*_ = *B*_*1*_ (i.e., the payer is indifferent about investing in preventive versus curative technologies), the ratio between the justifiable price of a vaccine used for a routine and catch-up programme and the price of the treatment is:

$$P_{1}/P_{0}=sn_{c}\frac{1+r_{r}-\psi_{t-1}\left(r_{r}\right)}{1+n_{c}r_{r}-\psi_{t-1}\left(r_{r}\right)}\leq sn_{c}$$

Consistent with the findings of Baumann [8], we find that a vaccine’s economically justifiable price can be considerably lower than that of a treatment for diseases with low prevalence across cohorts (i.e., those with a small *sn*_*c*_). However, this is not generally true for more widely prevalent diseases.

### Revenues

The treatment generates annual sales of (*c*_*c*_*n*_*c*_ + *B*_*0*_), which is the sum of the cost savings generated by the drug and of the incremental budget the regulator is willing to pay to eradicate the disease.

The revenues are a function of the total cost of the disease *c*_*c*_*n*_*c*_ and are independent of *s* for the treatment and the vaccine. Therefore, if the economics of equation (5) are followed, the decision to invest in a vaccine rather than a drug does not depend on the prevalence of the disease, but on the total cost of the disease. However, if we relax the assumptions of constant margins and costs, this conclusion may no longer be valid (see section "Impact of key assumptions").

If there is only one sick cohort (i.e., *n*_*c*_ = 1), the revenues are the same for all scenarios.

#### Routine plus catch-up versus treatment

The ratio between the revenues generated by the routine plus catch-up vaccination and those generated by the treatment is:

$$\begin{array}{l} \left[\frac{1-\psi_{t}\left(r_{r}\right)}{1-\psi_{t}\left(r_{m}\right)}\;\frac{1+r_{r}}{1+r_{m}}\;\frac{1+n_{c}r_{m}-\psi_{t-1}\left(r_{m}\right)}{1+n_{c}r_{r}-\psi_{t-1}\left(r_{r}\right)}\right]\;\frac{c_{c}n_{c}+B_{1}}{c_{c}n_{c}+B_{0}}\\ \;=\left[\frac{1+r_{r}-\psi_{t-1}\left(r_{r}\right)}{1+n_{c}r_{r}-\psi_{t-1}\left(r_{r}\right)}\;\frac{1+n_{c}r_{m}-\psi_{t-1}\left(r_{m}\right)}{1+r_{m}-\psi_{t-1}\left(r_{m}\right)}\right]\;\frac{c_{c}n_{c}+B_{1}}{c_{c}n_{c}+B_{0}} \end{array}$$

The ratio decreases with *r*_*r*_ and increases with *r*_*m*_, and is composed of two parts: (i) ratios of functions of the manufacturer and regulator discount rates; and (ii) the ratio of disease costs and budgets. It is worth noting that the revenues are identical if the regulator is indifferent about treatment versus prevention (i.e., *B*_*1*_ = *B*_*0*_) and if the manufacturer and regulator use the same discount rate (i.e., *r*_*r*_ = *r*_*m*_). If *B*_*1*_ > *B*_*0*_ and *r*_*m*_ > *r*_*r*_, the revenues from routine plus catch-up vaccination are higher than those from the treatment. This is because the relative value of the sales in the first year versus total sales is more important for the manufacturer than for the regulator if the manufacturer applies a higher discount rate. If *t* becomes large, the ratio approaches:

$$\frac{1+r_{r}}{1+r_{m}}\;\frac{1+n_{c}r_{m}}{1+n_{c}r_{r}}\;\frac{c_{c}n_{c}+B_{1}}{c_{c}n_{c}+B_{0}}$$

The ratio between the first-year revenues for routine plus catch-up vaccination versus those for treatment is:

$$\frac{1+r_{r}-\psi_{t-1}\left(r_{r}\right)}{1+n_{c}r_{r}-\psi_{t-1}\left(r_{r}\right)}\;\frac{c_{c}n_{c}+B_{1}}{c_{c}n_{c}+B_{0}}\;n_{c}$$

If *B*_*1*_ = *B*_*0*_, this leads to an asymptotic value in *t* of $n_{c}\frac{1+r_{r}}{1+n_{c}r_{r}}$; in this case, the upfront investment for routine plus catch-up vaccination can be large compared with that for the treatment and may be a barrier to implementation for the payer. If the number of impacted cohorts (*n*_*c*_) is small, the ratio is only slightly sensitive to the discount rate used by the regulator (*r*_*r*_). If the disease impacts a large number of cohorts, a small decrease in discount rate increases substantially the first year cost of the catch-up, relative to treatment.

#### Routine vaccine (without catch-up) versus treatment

As discussed, the price of the routine vaccination increases when *t* increases. However, even if *t* is large, the revenues from routine vaccination are lower than those from treatment. If the new technologies have to be cost neutral (i.e., *B*_*0*_ = *B*_*1*_ = 0), the discounted sales from routine vaccination are asymptotically $\frac{\left(n_{c}-1\right)}{2}\;r_{r}$ times lower than for treatment (as per Proposition 6 in Additional file 1). In other words, if *t* is large, routine vaccination becomes relatively less attractive than treatment as the number of cohorts *n*_*c*_ and the regulator discount rate *r*_*r*_ increase. This result was expected: the relative value of routine vaccination decreases as the number of cohorts increases because it takes many years to eradicate the disease. The regulator could largely eliminate this bias against routine vaccination by choosing a discount rate close to 0.

It is worth noting that if the technologies are expected to be cost neutral (i.e. if *B*_*0*_ = *B*_*1*_ = 0), the relative value of each scenario’s revenue is independent of *c*_*c*_. In other words, the annual cost of the disease per cohort is just a normalizing factor in this case.

### Numerical illustration

In order to quantify and illustrate the incentives described in the previous section, a base case was constructed as follows: the disease impacts 10 cohorts (i.e., *n*_*c*_ = 10), and the annual probability of becoming sick *s* = 1%. The regulator is not willing to spend additional budget on either the vaccine or the treatment (*B*_*0*_ *= B*_*1*_ = 0), i.e., new technologies have to be cost neutral. A discount rate *r*_*r*_ of 3% was used for the regulator, as recommended by the World Health Organization [10]. A discount rate *r*_*m*_ of 8% was used for the manufacturer [11]. The time horizon used for evaluation was 20 years (i.e., *t* = 20). Similar to the previous model, each cohort size is normalized to 1 (or 100%). The disease cost per cohort *c*_*c*_ was assumed to be $100.

Based on our model and the assumptions above, the maximal price of the treatment is $10,000 per individual, while the price of routine vaccination is $728 and the price of routine plus catch-up vaccination is $630 (only 6% of the treatment price). The discounted sales over 20 years of the treatment amount to $9818, those of the routine and catch-up vaccination are $11,435 and those of routine vaccination only are $7148 (i.e., 27% below those of the treatment). Therefore, the vaccine price in our numerical example is considerably below that of a drug, and the revenues from a routine vaccination are lower than from the treatment. However, if the regulator decides to implement a catch-up campaign, the revenues from the vaccine become more attractive to the manufacturer than those from the treatment.

The revenues in the first year are $1000 for the treatment, and $6300 for the routine and catch-up vaccination, compared with $728 for the routine vaccination alone. Therefore, the first-year budget impact of a routine and catch-up programme can be seen as prohibitive if the payer has not properly projected the budget needed for introducing the new vaccine.

#### Sensitivity analysis

**Figure 1:**
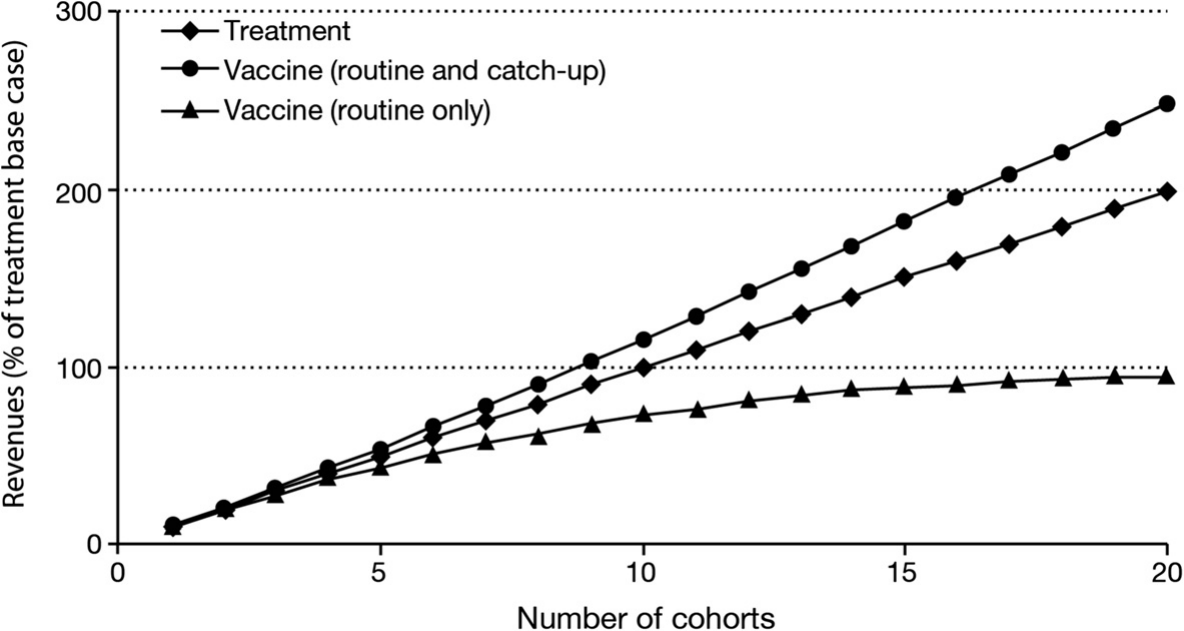
**Impact of the number of cohorts (*n***_***c***_**) on the sales potential.** 100% represents the discounted sales generated by the treatment for base case values (e.g., with *n*_*c*_ = 10).

**Figure 2:**
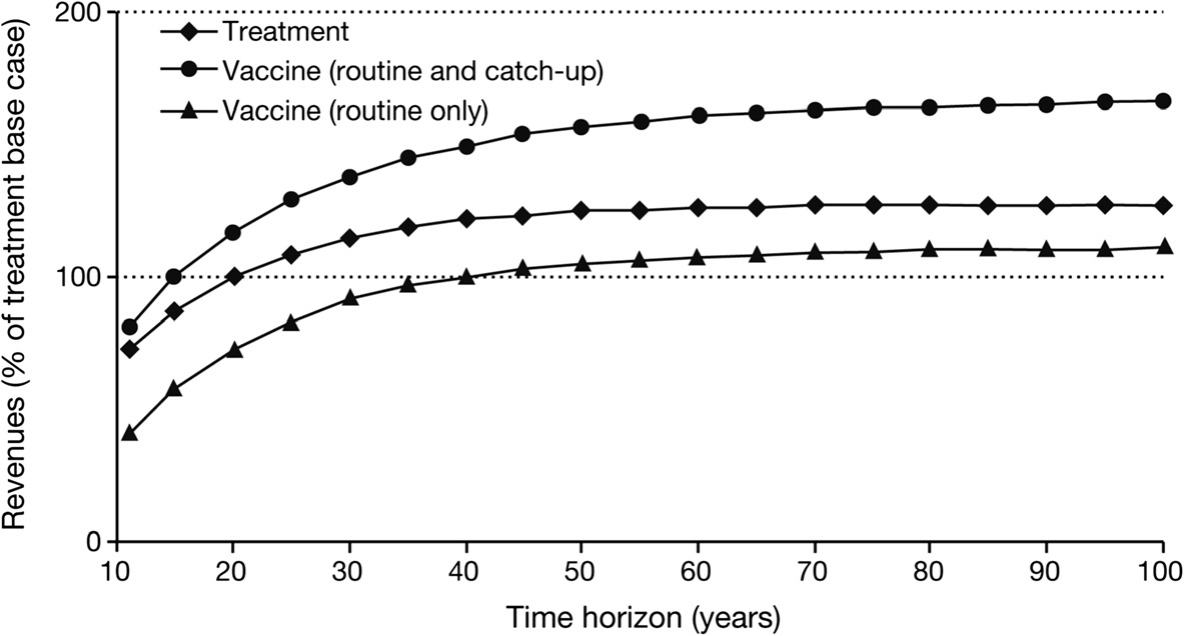
**Impact of the time horizon (*t*).** 100% represents the discounted sales generated by the treatment for base case values (e.g., with *t* = 20).

Univariate sensitivity analyses around the base case were conducted to assess the impact of key assumptions on the decision-making process. In particular, we modified the number of cohorts impacted by the disease (*n*_*c*_) while keeping all other assumptions fixed (including the cost per cohort) (see Figure 1). We concluded that the more widespread the disease, the less favourable the revenue associated with routine vaccination compared to curative alternatives. For instance, if the disease affects 20 cohorts, the sales potential of the routine vaccination is only half that of the treatment. Conversely, the larger the number of affected cohorts, the more favourable is the approach of a vaccination with catch-up, as the revenue in the first year will be very large. By increasing the time horizon, the disadvantage of the routine programme decreases. If *t* = 100, the difference in revenues decreases to 13% (versus 27% in the base case), which is close to the asymptotic value of $\frac{\left(n_{c}-1\right)}{2}\;r_{r}=14\%$ discussed in the section "Routine vaccine (without catch-up) versus treatment" (see Figure 2).

### Impact of key assumptions

The validity of some key assumptions and the impact of relaxing them should be considered.

#### Gross margins

In the above model, it was assumed that gross margins were the same for all technologies. In other words, $\frac{\left(P_{j}-c_{j}\right)}{P_{j}}$ (where *c*_*j*_ is the average cost of producing a unit of *j* over the evaluation period) is the same for *j* = 0 and *j* = 1. A number of considerations should be taken into account when assessing the validity of this assumption. Margins for vaccines could be lower than those for drugs: (i) because vaccine production is a more complicated (and potentially more costly) process than drug production because vaccines are produced from living organisms that must be grown in a highly controlled environment [1], and (ii) because prices of vaccines are lower than those of drugs (see "Price" section). Conversely, the large economies of scale (because vaccination affects entire cohorts and treatment affects only the percentage *s* of cohorts) could increase margins for vaccines. Therefore, it is possible – but not likely – that margins for drugs and vaccines are identical. If this is not the case, the manufacturer’s decision criteria will not be based on comparing discounted revenues as per equation (5), but on discounted revenues minus the cost of goods, which are:

(6) $$\frac{1-\psi_{t}\left(r_{m}\right)}{r_{m}}\;s\;c_{0}\;n_{c}$$

for the treatment, and:

(7) $$\frac{1-\psi_{t}\left(r_{m}\right)}{r_{m}}\;c_{1}$$

for the routine vaccination. Therefore if (8), the routine vaccination will be further penalized in the assessment by the regulator. Equation (8) can be interpreted as the economy of scale necessary to help justify investment in a vaccine. The increase in production should lead to a decrease in production costs large enough to ensure that the vaccine cost per unit is less than *sn*_*c*_ (number of sick individuals) times the cost of the treatment.

#### Development costs

Our assumption that development costs for vaccines and treatments are similar is justified by empirical data. In 2002, the cost of developing and licensing a vaccine was estimated to reach $700 million [12], which is within the range of that for drugs ($403–$802 million) [13].

#### Marketing and selling costs

The implied assumption in our model is that the drug and vaccines have the same procurement system and the same professional audience for promotion. If one treatment is sold via detailing to physicians while the other is purchased centrally by the regulator, this assumption will not hold true.

#### Time horizon

It was assumed that pharmaceutical companies had a time horizon *t* that exceeded *n*_*c*_. However, if *n*_*c*_ is large, this assumption may not be valid and that will further reduce the value of routine vaccination.

#### Factors not taken into account in the model

Our simplified model did not take into account certain parameters in the decision-making.

In our model, it was assumed that all eligible individuals could be vaccinated or treated; however, the vaccination and diagnosis rates could be less than 100% and their relative values will influence the investment decision. As in the case of the cost of goods, the relative value of fees for vaccine administration and for diagnosis could also impact on the price acceptable to the regulator and the investment decision. In addition, the efficacy of vaccines tends to decrease over time; for example, some vaccines, such as meningococcal and pertussis vaccines, are known to have low persistence [14,15]. If waning is assumed, the relative value of vaccines will further decrease.

Vaccination could have additional benefits. If vaccinated individuals prevent the spread of a disease via herd protection, not all susceptible cohorts have to be fully vaccinated to eliminate the disease, leading to a higher economic value of vaccination. The indirect effect (and the duration of protection) can have a substantial impact on vaccines’ economically justifiable price [16]. However, herd immunity can only be proven when a large number of individuals are vaccinated. Often, herd immunity is not shown when health authorities recommend a vaccine and implement a vaccination program under systematic surveillance. Therefore, health authorities may or may not assume indirect effects when making a health economic evaluation. For instance, the Joint Committee on Vaccination and Immunisation, when making its interim decision on vaccination against meningitis B, concluded that “current [indirect effects] evidence [was] insufficient to support a recommendation for the introduction of a routine adolescent immunisation programme” [17].

### Empirical evidence

#### Incentives to develop HIV interventions

In the previous sections, we developed a theoretical model to understand the incentives to invest in curative vs. preventive interventions. In this section, the model findings are applied to understand whether economic evaluation methods may (partially) explain the absence of a vaccine to prevent HIV. Numerous reasons to explain this absence have been mentioned in the literature. Scientific challenges are significant [18] but, according to Cohen [19] and Thomas [20], critical causes also include scientific infighting, the lack of co-ordination between institutions, and the lack of funding from pharmaceutical companies. In fact, Harris [21] estimated that in 2008, pharmaceutical companies only invested $33 million in research for an HIV vaccine. For Craddock [22], this inadequate research investment is “because the countries hardest hit by AIDS cannot afford to buy vaccines in quantities adequate to achieve a minimum profit margin”. However, worldwide sales of the HIV therapies ^a^ recommended by the US Department of Health and Human Services amounted to over $14 billion in 2010 [23]. Therefore, the HIV market cannot be considered unattractive for pharmaceutical companies: some other market incentives must explain the lack of a vaccine.

An alternative argument [21] is that governments will prevent price discrimination between high- and low-income countries, but this argument also applies to preventive medicines and does not specifically disadvantage vaccines. However, it is possible to deduce from Harris [21] that the HIV market is not unattractive to vaccine manufacturers per se, but that instead it is unattractive with respect to vaccines *as compared with* drugs. To test this, we can analyse the HIV market in the US using our framework described above.

As 86% of newly diagnosed HIV cases occur in people aged between 20 and 54 years, we can assume that *n*_*c*_ ≈ 35 (see Table 3). Using the discount rates from section "Numerical illustration" and assuming that *t* = *n*_*c*_, the expected revenues from routine vaccination of adolescents will be only 43% of those of a cure. As noted by Harris [21], the prospect of a large catch-up campaign that would favour a vaccine investment seems too unpredictable to motivate substantial R&D investment. If the time horizon used by pharmaceutical companies is only 10 years, the incentive to develop a routine vaccine will even be lower. The annual HIV incidence is approximately 1150 for each 1-year age cohort group (Table 3). Using US population tables, the incidence *s* is approximately 27 per 100 000 [24], and *n*_*c*_ *· s* < <1. Therefore, the economies of scale have to be very large to help justify a vaccine investment (i.e., *c*_1_ <<*c*_0_).

**Table 3:** Estimated^*^ number of diagnoses of HIV infection in 2010 [25]

**Age at diagnosis (years)**	Estimated^*^number of diagnoses	**Percentage of all diagnoses**
<13	217	<0.5
13–14	34	<0.1
15–19	2200	5
20–24	7565	16
25–29	6823	14
30–34	5954	13
35–39	5523	12
40–44	5720	12
45–49	5296	11
50–54	3671	8
55–59	2154	5
60–64	1119	2
≥65	853	2

^*^Estimated numbers resulted from statistical adjustment that accounted for reporting delays and missing risk-factor information.

### Scope and use of the model framework

This article is a theoretical article that could apply for diseases not yet preventable by immunization. Those include (but are not limited to) sexually transmitted diseases (AIDS, chlamydia, gonorrhoea, syphilis, etc.), tuberculosis, malaria, enterotoxic *escherichia coli*, respiratory syncytial virus, group B streptococcus, cytomegalovirus, etc.

The authors could not identify any company that developed a drug over a vaccine (or a vaccine over a drug) within the context of this model, potentially because companies do usually disclose major investment decision but keep the underlying strategic reasons and discussions confidential.

#### Recommendations for the regulator

Rappuoli et al. [5] and Lattanzi & Rappuoli [26] discussed a number of incentives to favour investment in vaccines. For instance, they recommended that vaccine development should benefit from tax breaks, extension of patent terms, creative use of the orphan drug law, reduction in liability risks, public–private partnerships, etc. Our paper highlights potential additional means to increase the incentives to invest in vaccines. In particular, the regulator could: (i) Choose a long-term horizon when evaluating health technologies (i.e., a high value for *t*). (ii) Choose a much lower discount rate compared with that used by the manufacturer. (iii) Conduct a catch-up campaign. (iv) Choose a lower discount rate for vaccines than for drugs. (v) Ensure that *B*_*1*_ exceeds *B*_*0*_. For regulators using budget-impact models, this means that additional budget should be allocated to vaccines. For regulators using a cost-effectiveness approach, it means that vaccines could be assigned a higher cost-effectiveness threshold to account for the intangible value of vaccines discussed by Masignani et al. [4]. In particular, the impact on productivity for the society as whole [4], utility by anticipation, and the impact on other countries could be taken into account in the evaluation. (vi) Ensure that the costs of developing, producing, and selling the vaccines are lower than those of drugs. This could take the form of central procurement with a guaranteed price to reduce selling costs. Similar to the suggestion of Berman and Giffin [1], it could also be a public–private partnership, such as a grant to develop the vaccine or to finance a manufacturing facility. The US government followed that path by awarding $60 million over 4 years to Novartis Vaccines and Diagnostics to expand their laboratory facilities and to include a pandemic influenza and emerging-disease centre [27].

The credibility of the regulator’s commitment is critical in the process. If this commitment is not perceived as credible by manufacturers, it may not foster vaccine innovation. However, as it takes approximately 10–12 years to bring a vaccine to market, the regulator’s commitment needs to be made when the development of the vaccine actually starts. Finally, considering budget cuts, health authorities may be reluctant to engage in large catch-up campaigns once a vaccine is approved.

## Conclusions

If no catch-up vaccination campaign is implemented, the expected value to the manufacturer of developing a vaccine is much lower than that of developing a drug, everything else being equal. This can be explained by the fact that with vaccination there is a ramp-up time required to achieve protection of the full population. However, if a catch-up campaign vaccinates all susceptible individuals, the expected revenues from vaccines could exceed those of a treatment (assuming there is no waning in efficacy). If the cost of goods is high for vaccines relative to drugs, vaccination is not likely to be as attractive for manufacturers as a curative treatment would be. This article provides additional tools to those already proposed by academics to increase incentives to invest in vaccines. However, we do not suggest that changing market incentives will necessarily lead to vaccines being developed against all relevant diseases, including HIV. Even within the current evaluation framework, vaccines against relatively rare diseases such as meningitis have been developed, suggesting that existing incentives may already be attractive to some manufacturers. Market incentives do not seem to be the only driving factor; other considerations such as clinical or humanistic arguments are usually also taken into consideration. The probability of clinical success for each type of intervention is undoubtedly crucial: for example, in rapidly progressing diseases in which the onset of the effect of treatment may be too slow once the disease is diagnosed, prevention may be viewed as a better strategy than treatment. Fast-progressing diseases, such as meningitis, may therefore be optimal candidates for a preventative vaccination programme.

## Endnote

^a^Drugs with sales above $100 million in 2010: Atripla ($2927 million), Truvada ($2746 million), Reyataz ($1479 million), Kaletra ($1255 million), Isentress ($1090 million), Prezista ($888 million), Epzicom ($858 million), Combivir ($561 million), Norvir ($344 million), Sustiva ($315 million), Viramune ($295 million), Intelence ($243 million), Trizivir ($223 million), Crixivan ($206 million), Epivir ($178 million), Ziagen ($159 million), Selzentry ($124 million), Viracept ($112 million); values from EvaluatePharma [23].

## Competing interests

The authors are employees of Novartis Vaccines & Diagnostics AG. This paper represents the view of the authors and should not be considered as representative of the view of Novartis Vaccines & Diagnostics AG.

## Authors’ contributions

SR conceived and developed the model and drafted the manuscript. JH contributed to the design of the model and the drafting of the paper. Both authors have read and approved the final manuscript.

## Authors’ information

The study reported in this paper was conducted as part of the author’s research at the University of Neuchâtel, Switzerland. The author and the co-author are employed by Novartis Vaccines & Diagnostics AG (NVD).

## Supplementary Material

Additional file 1 **Appendix with propositions and mathematical proofs.** This appendix provides mathematical proofs for propositions used in the model. Click here for file
