# Supplementary material for: Drug versus vaccine investment: a modelled comparison of economic incentives
Source: Cost Eff Resour Alloc. 2013 Sep 8;11:23. doi: 10.1186/1478-7547-11-23 (PMC3846654; doi:10.1186/1478-7547-11-23)
Supplement: Additional file 1 — Appendix with propositions and mathematical proofs. This appendix provides mathematical proofs for propositions used in the model. [file 1478-7547-11-23-S1.pdf]

Drug versus vaccine investment: a modelled comparison of economic incentives

Stephane Régnier and Jasper Huels

Additional file 1: Appendix with propositions and mathematical proofs

This appendix provides mathematical proofs for propositions used in the model.

Proposition 1

$$S_t = \sum_{i=1}^{t} \frac{1}{(1+r)^i} = \frac{1-\psi_t(r)}{r}$$

Proof:

$$S_t = \sum_{i=1}^{t} q^i = q \cdot \frac{1-q^t}{1-q}$$

If we replace q with $\frac{1}{1+r}$ we get:

$$S_t = \frac{1}{1+r} \cdot \frac{1 - {}^{1}\!/_{(1+r)^t}}{1 - {}^{1}\!/_{(1+r)}} = \frac{1-\psi_t(r)}{r}$$

Proposition 2

$$S_t = \sum_{i=1}^{t} iq^i = \frac{q-(t+1)q^{t+1}+tq^{t+2}}{(1-q)^2}$$

Proof:

$$S_t - qS_t = (1-q)S_t = \sum_{i=1}^{t} iq^i - \sum_{i=1}^{t} iq^{i+1} = \sum_{i=1}^{t} iq^i - \sum_{i=2}^{t+1}(i-1)q^i = \sum_{i=1}^{t} iq^i - \sum_{i=1}^{t+1}(i-1)q^i = \sum_{i=1}^{t} q^i - tq^{t+1} = q\frac{1-q^t}{(1-q)} - tq^{t+1} = \frac{q-(t+1)q^{t+1}+tq^{t+2}}{(1-q)}$$

$$\Rightarrow S_t = \frac{q-(t+1)q^{t+1}+tq^{t+2}}{(1-q)^2}$$

Proposition 3

$$S_t = \sum_{i=1}^{n_c} i \frac{1}{(1+r)^i} + \sum_{i=n_c+1}^{t} n_c \frac{1}{(1+r)^i} = \frac{1+r-\psi_{n_c-1}(r) - n_c r \psi_t(r)}{r^2}$$

Proof:

Considering the following series:

$$T_t = \sum_{i=1}^{n_c} i q^i + \sum_{i=n_c+1}^{t} n_c q^i$$

$$T_t - qT_t = \sum_{i=1}^{n_c} iq^i - \sum_{i=1}^{n_c} iq^{i+1} + \sum_{i=n_c+1}^{t} n_c q^i - \sum_{i=n_c+1}^{t} n_c q^{i+1} = \sum_{i=1}^{n_c} iq^i - \sum_{i=2}^{n_c+1} (i-1)q^i + \sum_{i=n_c+1}^{t} n_c q^i - \sum_{i=n_c+2}^{t+1} n_c q^i = \sum_{i=1}^{n_c} q^i - n_c q^{n_c+1} + n_c q^{n_c+1} - n_c q^{t+1} = q\frac{1-q^{n_c}}{(1-q)} - n_c q^{t+1} = \frac{q - q^{n_c+1} - (1-q) n_c q^{t+1}}{(1-q)}$$

$$\Rightarrow T_t = \frac{q - q^{n_c+1} - (1-q) n_c q^{t+1}}{(1-q)^2}$$

If we replace q by $\frac{1}{1+r}$ we get:

$$S_t = \frac{1/(1+r) - 1/(1+r)^{n_c+1} - (1 - 1/(1+r)) \cdot n_c \cdot 1/(1+r)^{t+1}}{(1 - 1/(1+r))^2} = \frac{1+r - 1/(1+r)^{n_c-1} - r \cdot n_c \cdot 1/(1+r)^t}{r^2} = \frac{1+r-\psi_{n_c-1}(r) - r \cdot n_c \cdot \psi_t(r)}{r^2}$$

Proposition 4a

The maximal achievable price for routine immunization combined with catch-up in the first year is:

$$P_1 = \frac{1 + r_r - \psi_{t-1}(r_r)}{1 + n_c r_r - \psi_{t-1}(r_r)} \cdot (c_c n_c + B_1)$$

Proof:

$Q_{1i} = n_c$ if $i = 1$ and $Q_{1i} = 1$ if $i > 1$, $CA_{1i} = c_c n_c$ which, by updating equation **(1)** leads to:

$$\frac{n_c P_1 - n_c c_c - B_1}{(1 + r_r)} + \sum_{i=2}^{t} \frac{P_1 - n_c c_c - B_1}{(1 + r_r)^i} \leq 0 \text{ <=> } \frac{(n_c - 1)P_1}{(1 + r_r)} + \sum_{i=1}^{t} \frac{P_1 - n_c c_c - B_1}{(1 + r_r)^i} \leq 0$$

From Proposition 1:

$$\frac{(n_c - 1)P_1}{(1 + r_r)} + (P_1 - n_c c_c - B_1) \cdot \frac{1 - \psi_t(r_r)}{r_r} \leq 0$$

Multiplying with $(1 + r_r) \cdot r_r$ leads to:

$$(n_c - 1) \cdot r_r \cdot P_1 + P_1 \cdot (1 + r_r) \cdot (1 - \cdot \psi_t(r_r)) \leq (n_c c_c + B_1) \cdot (1 + r_r) \cdot (1 - \cdot \psi_t(r_r))$$

Since $(1 + r_r) \cdot (1 - \cdot \psi_t(r_r)) = (1 + r_r - \psi_{t-1}(r_r))$,

$$(n_c - 1) \cdot r_r \cdot P_1 + P_1 \cdot (1 + r_r - \psi_{t-1}(r_r)) \leq (n_c c_c + B_1) \cdot (1 + r_r - \psi_{t-1}(r_r))$$

The maximal acceptable price for the regulator is:

$$(n_c c_c + B_1) \cdot \frac{1 + r_r - \psi_{t-1}(r_r)}{1 + n_c r_r - \psi_{t-1}(r_r)}$$

Proposition 4b

The discounted revenue (from the manufacturer's perspective) for routine immunization combined with catch-up in the first year is:

$$\frac{1-\psi_t(r_r)}{r_m}\cdot\frac{1+r_r}{1+r_m}\cdot\frac{1+n_c r_m-\psi_{t-1}(r_m)}{1+n_c r_r-\psi_{t-1}(r_r)}\cdot(c_c n_c+B_1)$$

Proof:

$Q_{1i} = n_c$ if $i = 1$, otherwise $Q_{1i} = 1$

$$\sum_{i=1}^{t}\frac{Q_{1i}P_{1i}}{(1+r_m)^i}=\frac{n_c-1}{(1+r_m)}P_1+\sum_{i=1}^{t}\frac{P_1}{(1+r_m)^i}=P_1\cdot\frac{1+n_c r_m-r_m\psi_t(r_m)-\psi_t(r_m)}{r_m(1+r_m)}=P_1\cdot\frac{1+n_c r_m-\psi_{t-1}(r_m)}{r_m(1+r_m)}$$

$$=\frac{1+r_r-\psi_{t-1}(r_r)}{1+n_c r_r-\psi_{t-1}(r_r)}\cdot\frac{1+n_c r_m-\psi_{t-1}(r_m)}{r_m(1+r_m)}(c_c n_c+B_1)=\frac{(1+r_r)(1-\psi_t(r_r))}{r_m(1+r_m)}\cdot\frac{1+n_c r_m-\psi_{t-1}(r_m)}{1+n_c r_r-\psi_{t-1}(r_r)}\cdot(c_c n_c+B_1)$$

Proposition 5

The maximal price for routine immunization is:

$$P_1 = B_1 + c_c \frac{1 + r_r - \psi_{n_c-1}(r_r) - n_c r_r \psi_t(r_r)}{r_r(1 - \psi_t(r_r))}$$

Proof:

$Q_{1i} = 1$, $CA_{1i} = ic_c$ if $i \le n_c$ and $CA_{1i} = c_c n_c$ if $i > n_c$ leading to an updated equation **(4)**

$$\sum_{i=1}^{n_c} \frac{P_1 - ic_c - B_1}{(1+r_r)^i} + \sum_{i=n_c+1}^{t} \frac{P_1 - c_c n_c - B_1}{(1+r_r)^i} \le 0$$

which can be rewritten as:

$$\sum_{i=1}^{t} \frac{P_1 - B_1}{(1+r_r)^i} - \sum_{i=n_c+1}^{t} \frac{c_c n_c}{(1+r_r)^i} - \sum_{i=1}^{n_c} \frac{ic_c}{(1+r_r)^i} \le 0$$

By using Proposition 1 and Proposition 3:

$$(P_1 - B_1)\frac{1 - \psi_t(r_r)}{r_r} - c_c \frac{1 + r_r - \psi_{n_c-1}(r_r) - n_c r_r \psi_t(r_r)}{r_r^2} \le 0$$

Therefore, the maximal acceptable price is:

$$P_1 = B_1 + c_c \frac{1 + r_r - \psi_{n_c-1}(r_r) - n_c r_r \psi_t(r_r)}{r_r(1 - \psi_t(r_r))}$$

Proposition 6

If $B_1 = B_0 = 0$, the revenues from routine vaccination are asymptotically (i.e., when $t \to +\infty$)

$\frac{(n_c - 1)}{2} \cdot r_r$ lower than those of treatment.

Proof:

Asymptotically, the discounted revenues are:

$$D_0 = \frac{c_c n_c + B_0}{r_m} \text{ for treatment, and}$$

$$D_1^r = \frac{1}{r_m}(B_1 + c_c \frac{1 + r_r - \psi_{n_c - 1}(r_r)}{r_r}) \text{ for routine vaccination.}$$

Since $\psi_{n_c - 1}(r_r) = 1 - (n_c - 1) \cdot r_r + \frac{1}{2}(-n_c + 1).(-n_c) \cdot r_r^2 + o(r_r^3)$

then $D_1^r \sim_{t \to +\infty} \frac{1}{r_m}(B_1 + c_c n_c - \frac{1}{2}(n_c - 1) n_c c_c r_r)$ if r_r^2 is small.

Therefore, if r_r is small and $B_1 = B_0 = 0$:

$$\frac{D_1^r - D_0}{D_0} \sim_{t \to +\infty} -\frac{(n_c - 1)}{2} \cdot r_r$$
